# The effects of an 8-week stabilization exercise program on lumbar movement sense in patients with low back pain

**DOI:** 10.1186/s12891-016-0875-4

**Published:** 2016-01-14

**Authors:** Jean-Alexandre Boucher, Richard Preuss, Sharon M. Henry, Jean-Pierre Dumas, Christian Larivière

**Affiliations:** School of Rehabilitation, Faculty of Medicine, Université de Montréal, Montréal, Québec Canada; School of Physical & Occupational Therapy, McGill University, Montréal, Québec Canada; Deparment of Rehabilitation and Movement Science, The University of Vermont, Burlington, Vermont USA; School of Rehabilitation, Faculty of Medicine, Université de Sherbrooke, Sherbrooke, Québec Canada; Occupational Health and Safety Research Institute Robert-Sauvé (IRSST), Montréal, Québec Canada; Center for Interdisciplinary Research in Rehabilitation of Greater Montreal (CRIR), Montreal Rehabilitation Institute, Montreal, Québec Canada

**Keywords:** Proprioception, Movement sense, Lumbar spine, Low back pain, Stabilization exercise

## Abstract

**Background:**

Lumbar stabilization exercises have gained popularity and credibility in patients with non-acute low back pain. Previous research provides more support to strength/resistance and coordination/stabilisation programs. Some authors also suggest adding strength/resistance training following motor control exercises. However, the effect of such a lumbar stabilization program on lumbar proprioception has never been tested so far. The present study investigated the effects of an 8-week stabilization exercise program on lumbar proprioception in patients with low back pain (LBP) and assessed the 8-week test-retest reliability of lumbar proprioception in control subjects.

**Methods:**

Lumbar proprioception was measured before and after an 8-week lumbar stabilization exercise program for patients with LBP. Control subjects participated in the same protocol but received no treatment.

**Results:**

The lumbar proprioception measure showed moderate reliability. Patients with LBP and control subjects demonstrated no differences in lumbar proprioception at baseline. Participants from both groups showed better proprioception following the 8-week interval, demonstrating the presence of learning between testing days.

**Conclusions:**

The improvement of lumbar proprioception seen in both groups was ascribed to motor learning of the test itself. The effect of lumbar stabilization exercises on lumbar proprioception remains unknown because the LBP group did not show lumbar proprioception impairments.

**Electronic supplementary material:**

The online version of this article (doi:10.1186/s12891-016-0875-4) contains supplementary material, which is available to authorized users.

## Background

Systematic literature reviews indicate that physical exercises are effective in reducing pain and disability in patients suffering from low back pain (LBP) [1]. Although previous research has not succeeded in showing the superiority of one type of exercise program over another [2], a recent meta-analysis provides more support to strength/resistance and coordination/stabilisation programs [3].

Lumbar coordination/stabilization exercises are designed to restore the neuromuscular control of the lumbar spine. In Panjabi’s model of the stabilizing system of the spine [4], an impairment within the passive support system may lead to joint instability, and has to be compensated by the coordinated action of the spinal musculature. Panjabi’s model suggests that inadequate lumbar proprioception may contribute to impairments in the passive support system, and may also perpetuate the joint instability, leading to chronic pain [4].

Proprioception can be defined as the sense of stationary position and movement awareness [5]. In the spine, viscoelastic structures and muscles have mechanoreceptors that monitor trunk position and movement [6]. In patients with chronic LBP, altered central processing of mechanoreceptor afferent signals may lead to neuromuscular control deficits [7–9]. Paraspinal muscle atrophy [10] and increased fatigability [11] might also represent additional compromises to lumbar stability in patients with LBP. This may explain why some authors suggest adding strength/resistance training following motor control exercises [12]. The effect of such a lumbar stabilization program on lumbar proprioception has never been tested.

The first aim of this study was to determine the effects of an 8-week stabilization exercise program on lumbar proprioception in patients with LBP. A secondary aim was to assess the 8-week test-retest reliability of lumbar proprioception data, with no intervention, in control subjects. The control group was used for pre-treatment comparisons with the patients and to account for possible learning effects from the testing alone. It was hypothesized that patients with LBP would show initial lumbar proprioception impairments, compared to control subjects, that would disappear (or at least be reduced) at the end of the exercise program.

## Methods

### Participants

Sample size for the reliability study was estimated in order to obtain a target reliability coefficient (ICC) of 0.80 (95 % confidence interval: 0.6–1.0). Based on five experimental trials over two individual testing sessions, a minimum of 28 participants was needed considering the abovementioned requirements [13]. The study conducted by Lee et al. [14] is the only one reporting a statistically significant difference between both groups with regard to MPT in axial rotation, and using 24 patients with LBP and 24 control subjects. Based on the study conducted by Lee et al. [14] the present study have been conservative with a sample size of 29 patients with LBP and 30 control subjects. All participants were aged between 18 and 65 years old, and were recruited through newspaper advertisements and from physiotherapy clinics in Montreal, Quebec, Canada. All participants spoke French and English. Patients with LBP had lumbar or lumbosacral pain, with or without radicular pain, for at least four weeks (non-acute phase), and a score above 12 % on the Oswestry Disability Index (ODI) [15] to allow a minimal important change of 10 % [16] to occur. Patients with non-acute LBP were targeted because exercise is not a primary intervention strategy for acute LBP [1]. General exclusion criteria were: surgery of the pelvis or spinal column; a specific lumbar pathology (fracture, infection or tumor) or scoliosis; systemic or degenerative disease; body mass index over 30 kg/m^2^; having begun an exercise program in the last three months; pregnancy and claustrophobia. Additional exclusion criteria for patients with LBP were: having one positive neurological sign in two of three test categories: (1) Achilles and patellar tendon reflexes; (2) Reduced strength in myotomes; (3) Reduced sensation in dermatomes; and litigation relative to the back injury. Exclusion criteria for the control subjects were back pain in the preceding year or a history of back pain lasting more than 1 week.

Before testing, each participant was informed of all experimental procedures and provided their informed written consent. All procedures were approved by the ethics committees of the Centre for Interdisciplinary Research in Rehabilitation of Greater Montreal (CRIR) (ethical registration number: CRIR-738-0512).

### Lumbar stabilization program

An 8-week lumbar stabilization exercise program (two 30-min sessions/week) was provided to the patients with LBP in local physiotherapy clinics, without any co-intervention allowed. The patients were encouraged to do the exercises at home. Briefly, the exercise program focused on motor control of the deep trunk muscles [17], followed by gradual inclusion of overloading exercises designed to improve endurance and strength of the paraspinal and abdominal muscles [18] (further details in the Additional file 1).

### Questionnaires

The ODI was used to assess LBP-related disability [15] while the 11-point (0 to 10) numeric pain rating scale (NPRS) was used to assess the current, best and worst levels of pain intensity during the last week, so as to average the three ratings [19].

### Lumbar proprioception assessment

**Fig. 1:**
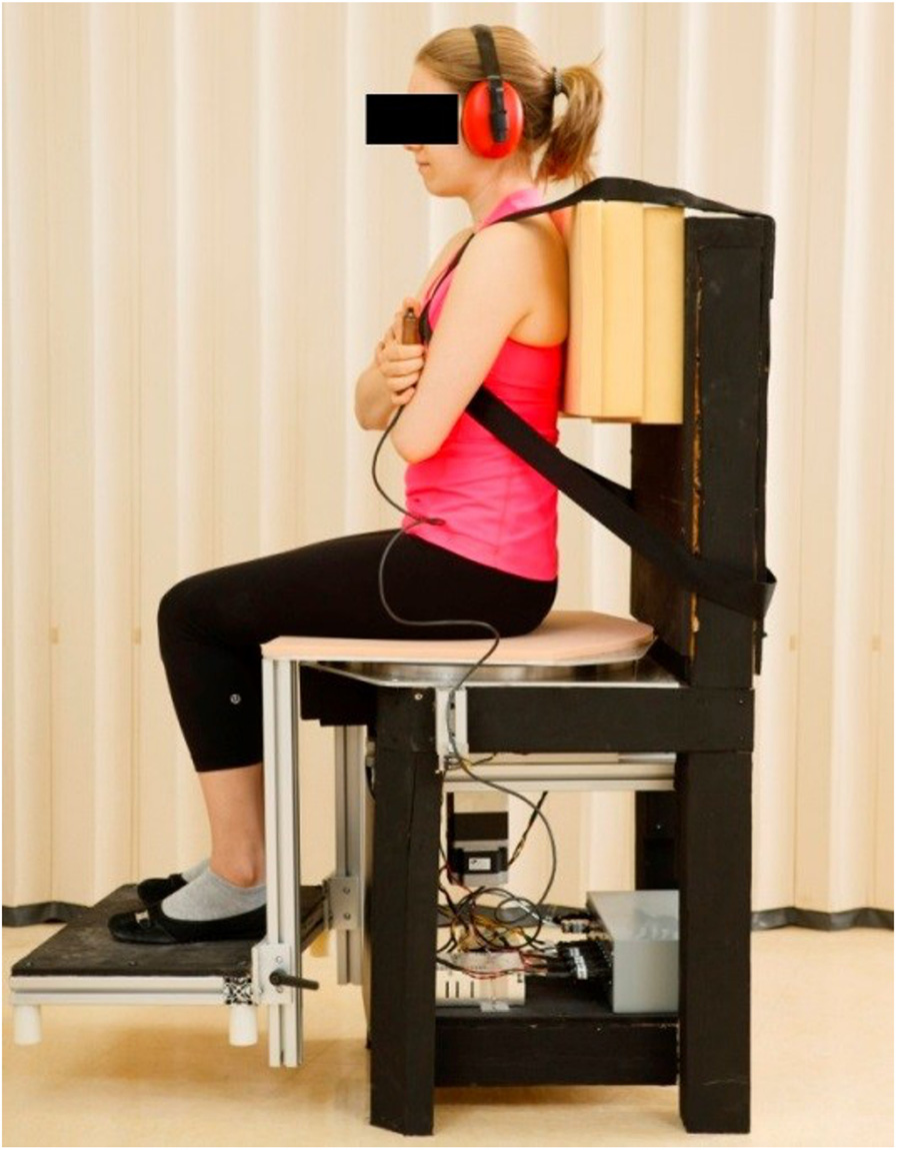
Motor driven lumbar proprioception chair. The upper body was fixed to the backrest while the lower body (pelvis and lower limbs) was rotated in the transverse plane, inducing an axial rotation of the lumbar spine. In this figure, the lower body is slightly rotated to the right

Lumbar motion sense, or motion perception threshold (MPT), was evaluated using a custom built apparatus similar to ones used in previous studies [14, 20] (Fig. 1). The MPT test measured the smallest amount of axial trunk rotation a participant could perceive. This measure was preferred over the measure of lumbar joint position sense because previous findings suggest that this test better discriminates between patients and control subjects [14], and produces more reliable data [20]. During the test, the lumbar spine is passively rotated in the transverse plane (trunk rotation) by rotating the lower body (seat). The seat is positioned on a 16-inches diameter high quality ball bearing (Silverthin Bearing Group, Preston, WA, USA; model SG160CPO) designed to minimize vibration. Movement is driven by a stepper motor (Applied Motion Products, Watsonville, CA, USA; model HT34-504) at a slow, steady rate to minimize tactile cueing. The resolution of the angular measurement was 0.09°.

Participants were positioned in the apparatus so that the L5/S1 joint aligned with the stepper motor shaft. The participant’s upper body was secured to the backrest with a 4-point seatbelt to minimize vestibular feedback. Starting from the neutral (zero) position, the seat was rotated, either clockwise or counter-clockwise, at a constant rate of 0.2°/s. As soon as motion was perceived, participants stopped the rotation by pressing a switch, and immediately stated the direction of movement. To remove trials where the subject would have guessed the motion perceptions, the trial was rejected if the direction perceived did not correspond to the true direction [14]. This was done in an attempt to reduce potential noise in the data. Participants were returned to the neutral position following each trial. All trials were performed with eyes closed and noise-canceling headphones.

Prior to testing, participants were given five familiarization trials (or more; until the task was properly understood), randomized for direction. This was followed by ten experimental trials also randomized for direction (five trials per direction).

All testing was done at the start of the study (session 1) and after 8 weeks (session 2).

### Home-exercise adherence

Home-exercise adherence was assessed with one question at the end of the 8-week clinical program: How many times have you done your exercises as prescribed in the last week? The frequency per week was divided by the recommendation of the physiotherapist to obtain a ratio, in accordance with the most common definition of adherence, which is defined as “the extent to which the patient follows medical instructions” [21]. The ratio may vary between 0 and 1, one being given when the frequency was equal or higher than prescription. The measurement of adherence was not carried out during the course of the 8-week clinical program to avoid influencing positively the adherence behavior (e.g. desirability bias).

### Data analysis

MPT scores were expressed as absolute values, in degrees. No significant differences between left and right rotations were detected, so the data were combined. Outliers (outside the 2-interquartile range) were removed from the 10 MPT scores, to reduce variability. The mean of MPT value, for each participant, was used for analysis.

#### Reliability statistical analyses

The reliability of the MPT data from the 30 control subjects was assessed within the generalizability theory framework [22], using a 2-way (2 DAY × 10 TRIALS) ANOVA for repeated measures. The computed sources of variance were used to calculate the dependability coefficients (ϕ) and standard error of measurement (SEM) [23]. D-study (decision study) results are reported, based on averaging data from 1, 5 and 10 trials within the same testing session (averaging across days is impractical), and may be interpreted in the same manner as an intraclass correlation coefficient [24].

#### Between-group and between-session statistical analyses

The healthy subjects whom participated to the reliability study were also used as a “control” group here. This allows testing whether the patients with LBP had proprioceptive deficits at baseline and also allows to estimate if systematic changes that could be detected during the treatment in the patients are attributable to the treatment of to the learning of the measurement protocol. MPT was assessed using a 3-way ANOVA (GROUP: LBP and control subjects; SEX: male and female; DAY: session 1 and 2). Significant interactions or main effects were further analyzed using a post hoc Tukey-Kramer test.

In patients with LBP, clinical outcome measures (NPRS, ODI) were assessed using a 2-way ANOVA (SEX; DAY). Partial Pearson’s correlations were also carried out between the change (session 2–session 1) of the MPT measure and the corresponding change of the clinical outcome measures, accounting for the baseline (session 1) MPT measure.

The analyses above were done with NCSS statistical software (version 8.0 for Windows), with the significance level set at *P* < 0.05.

## Results

Preliminary analyses revealed no group (LBP status), SEX or GROUP × SEX interaction for age, weight or height (Table 1). Among LBP patients, there was no significant SEX effect for NPRS and ODI (Table 1). The duration of pain was distributed as follows across the various intervals proposed [25]: (1) Less than 1 month (*n* = 0), (2) 1–3 months (*n* = 2), (3) 3–6 months (*n* = 1), (4) 6 months-1 year (*n* = 4), (5) 1–5 years (*n* = 10), (6) over 5 years (*n* = 12). The sample was thus constituted of 93.1 % (27/29) of patients with chronic pain (3 months or more).

**Table 1:** Demographic and clinical profiles [Mean (SD)] of the participants at initial measurements session (Day 1) according to their sex and clinical conditions

	Control subjects		LBP patients		*P* values		
	Men	Women	Men	Women	Group	Sex	Group × Sex
Variable	(*n* = 15)	(*n* = 15)	(*n* = 14)	(*n* = 15)			
Age (years)	39.3 (14.3)	39.8 (13.7)	43.5 (13.3)	47.3 (12.0)	0.093	0.424	0.620
Height (cm)	178.0 (8.4)	164.2 (6.1)	172.7 (5.9)	163.6 (5.3)	0.080	0.124	0.163
Weight (kg)	77.1 (10.7)	62.9 (10.9)	76.0 (13.3)	72.2 (9.6)	0.154	0.331	0.069
BMI (kg/m^2^)	24.4 (3.2)	23.3 (3.6)	25.7 (3.8)	25.4 (3.4)	0.063	0.466	0.249
NPRS (/10)			3.0 (2.2)	3.6 (1.5)		0.388	
ODI (%)			27.9 (9.2)	30.0 (9.8)		0.518	

*BMI* body mass index, *NPRS* numeric pain rating scale, *ODI* Oswestry Disability Index

### Reliability

**Fig. 2:**
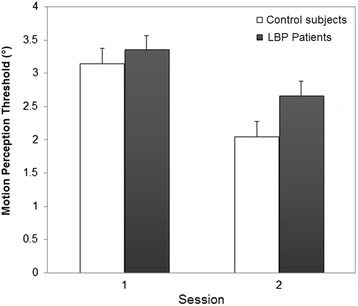
Averaged (across individuals) motion perception threshold (MPT) for both group in each testing session (mean ± SD)

For the control group, the 2-way ANOVA detected a DAY effect (*P* < 0.001) (Fig. 2), but no significant TRIAL effect (*P* = 0.833) or interaction (*P* = 0.663). The lack of a TRIAL effect indicates that no within-session learning was present, allowing us to average data across trials to increase reliability.

As expected, the D-study results revealed increased reliability (ϕ increase and SEM decrease) when averaging more trials (Table 2). Averaging 10 trials produced moderately reliable data (ϕ = 0.59 and SEM = 1.2°).

**Table 2:** Reliability results (D-Study)

Variable	Mean	Strategy: 1 trial/day			Strategy: 5 trials/day			Strategy: 10 trials/day			N ^a^
	(SD)	ϕ	SEM	SEM(%)	ϕ	SEM	SEM(%)	ϕ	SEM	SEM(%)	ϕ > 0.75
MPT	2.6 (2.2)	0.43	1.7	66	0.57	1.3	50	0.59	1.2	47	∞

*SD* Standard Deviation, *ϕ* Index of dependability, *SEM* standard error of measurement, *SEM (%)* SEM expressed in percentage of the grand mean; ^a^ Number of trials required to reach ϕ ≥ 0.75, ∞ indicating when it is impossible to reach this threshold

For the between-groups comparison (above), seven, eight, nine or ten trials were averaged per session (representing 2, 8, 18 and 72 % of the testing sessions, respectively), after having rejected the outliers. Consequently, the data used in the between-groups comparison are likely to be moderately reliable.

### Effects of the stabilization exercise program and between-group comparisons

**Fig. 3:**
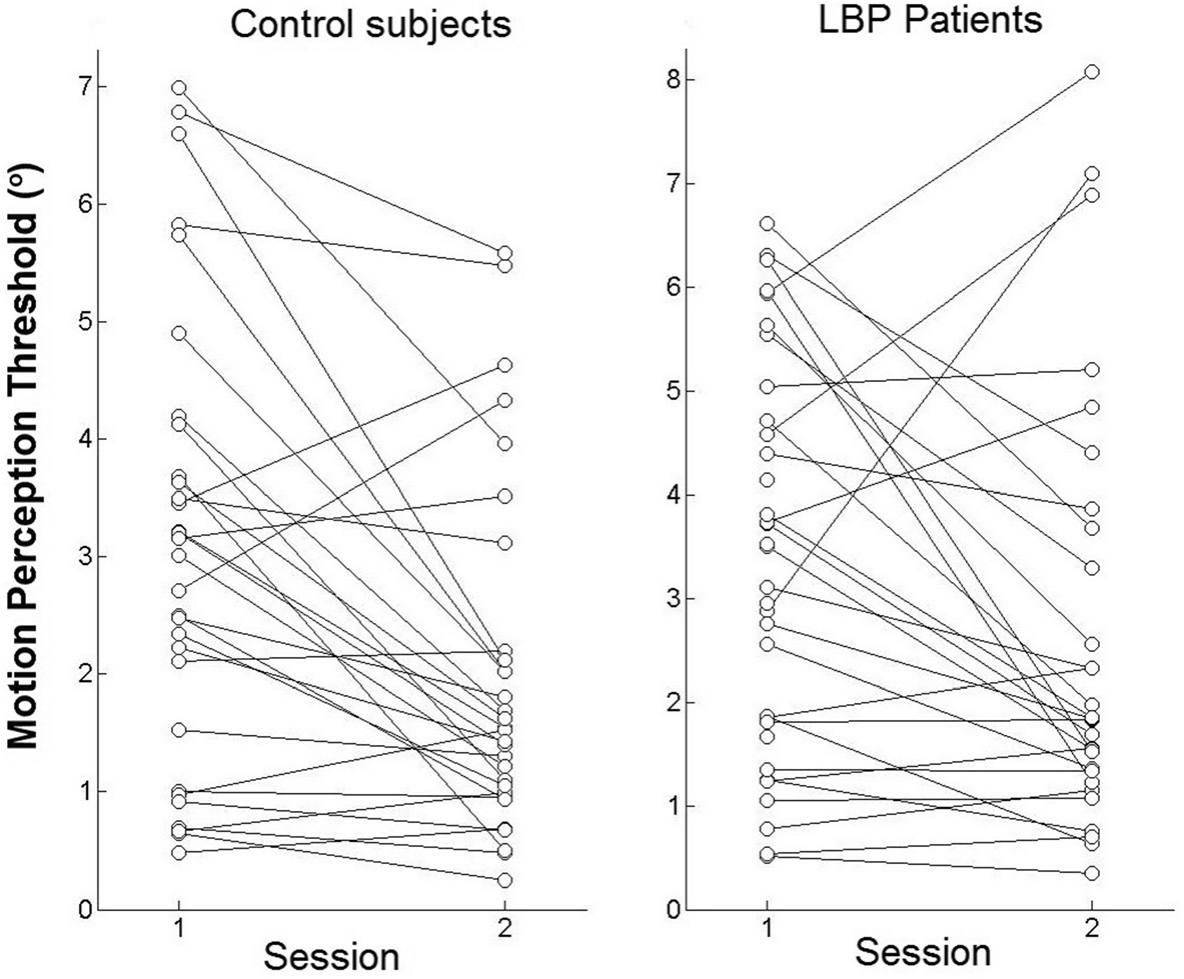
Line graph representing MPT scores of all participants in each group for both testing sessions

All patients with LBP have undertaken the 8-week exercise program and reached the third (final) phase of the exercise program. Home-exercise adherence measured at post-treatment was high, with a mean ratio of 0.86 (SD 0.21). For MPT, the only significant finding was a main effect of DAY (*P* < 0.001) (Table 3), showing significantly smaller MPT values at the second testing session (Fig. 2). The lack of a GROUP × DAY interaction reveals that the change in MPT measures over the 8-week period was not significantly different between the two groups. Figure 3 shows MPT scores of each participant for both testing sessions. The variability of the scores and variability of effects (time/treatment) were similar between groups.

**Table 3:** Statistical results (*P* values) corresponding to the comparisons between control subjects and patients with LBP (GROUP factor), between men and women (SEX factor), between measurement sessions (DAY factor) and the corresponding interactions

Variable	ANOVA results (*P* values)						
	GROUP	SEX	DAY	G × S	G × D	S × D	G × S × D
	(G)	(S)	(D)				
NPRS	/	0.641	<0.001	/	/	0.513	/
ODI	/	0.429	<0.001	/	/	0.547	/
MPT	0.239	0.509	<0.001	0.303	0.271	0.253	0.088

*NPRS* numeric pain rating scale, *ODI* Oswestry Disability Index, *MPT* motion perception threshold; “/” = not applicable because the corresponding ANOVA model did not include a GROUP factor

**Fig. 4:**
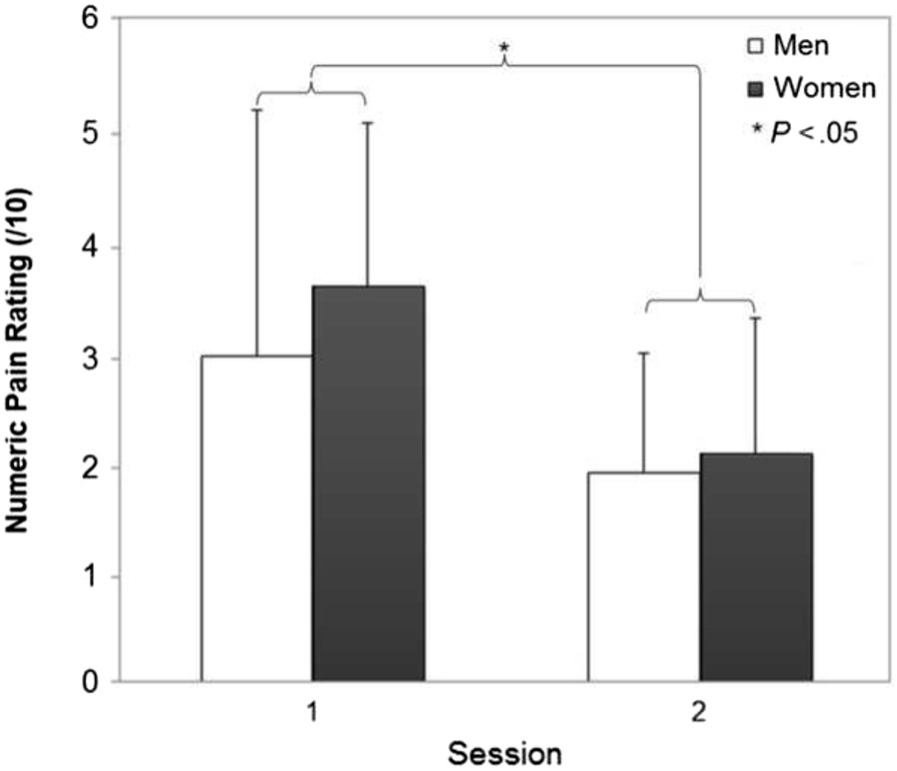
Numeric pain rating scale (NPRS) for each sex and testing day (mean ± SD)

**Fig. 5:**
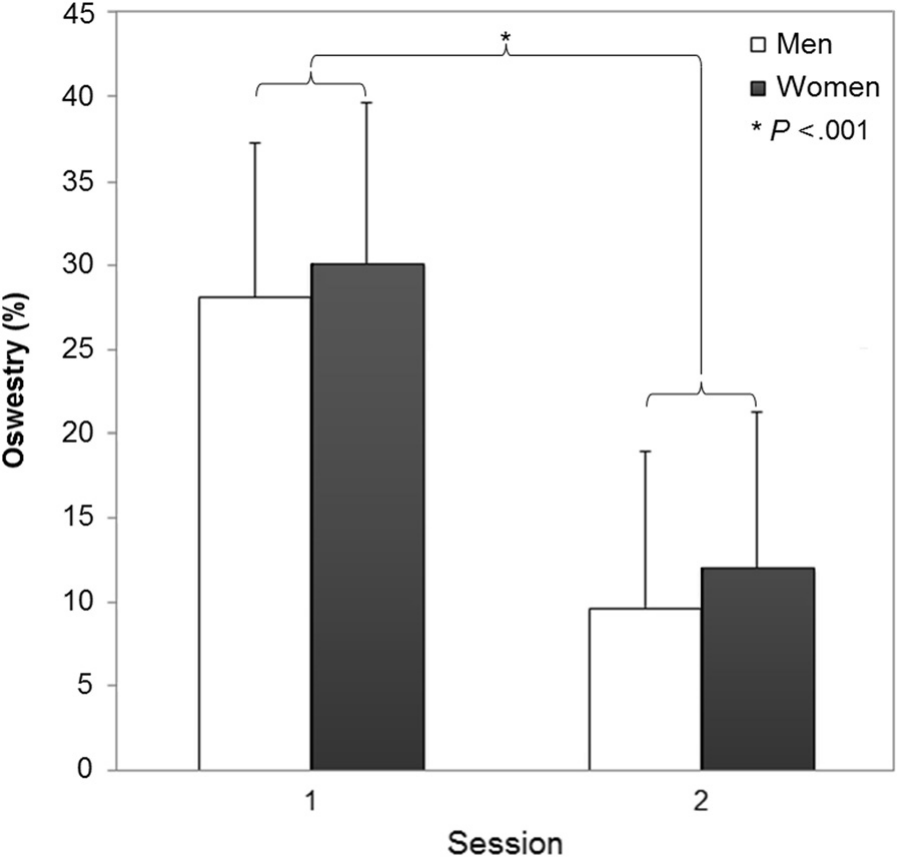
Oswestry (ODI) disability scores for each sex and testing day (mean ± SD)

A significant effect of DAY was also found for the clinical measures (NPRS, ODI) in patients with LBP, showing a decrease of pain and disability after the stabilization program (Table 3; Figs. 4 and 5). No significant SEX × DAY or SEX effects were found.

Partial correlation analyses revealed no significant correlations between changes in MPT scores and changes in NPRS (r = −0.06; *P* = 0.744) or ODI (*r* = −0.10; *P* = 0.602).

## Discussion

The primary aim of the present study was to assess the effects of an 8-week stabilization exercise program on the lumbar MPT in patients with LBP. While the patients showed a lower MPT at the end ot the stabilization program, a similar effect was found in control subjects, for whom no treatment was provided. In fact, no between-group differences were found. In line with these findings, no significant correlation was found between the change of MPT and the change of LBP intensity (NPRS) or LBP-related disability (ODI).

The secondary aim of the study was to assess the 8-week test-retest reliability of MPT in control subjects. The reliability was moderate, and illustrated the presence of a systematic learning effect between testing sessions.

Before discussing these findings, it is important to explain why the MPT scores reported here are much higher than the scores of the studies that have used a similar protocol and device [14, 20], the averaged scores (across individuals) ranging between 1.1 and 1.4° in these studies. In these studies, the two worst trials (highest scores) were removed from their 8 to 10 trials available, while in the present study, only outliers (worst or best scores) were removed. This may at least partly explain our higher averaged scores, the number of practice trials being approximately the same.

### 8-week test-retest reliability

The present reliability findings are the first attempt to assess the reliability of MPT data over the duration of a rehabilitation program (8 weeks). Two previous studies have reported reliability results for MPT measures taken with a device similar to the one used in the present study [8, 20]. The reliability of these previous data were high (ICCs over 0.77; SEM: 0.34°), but only permitted a within-day reliability analysis. The lower reliability of our data are at least partly explained by an apparent long-term learning effect (main effect of DAY), which suggest the presence of motor learning. The familiarization trials that were used at the beginning of each day were apparently good enough to eliminate possible within-session learning (no TRIAL main effect), but insufficient to eliminate between-day learning, the latter being likely explained by the effect of sleep on neural plasticity [26]. This is worth mentioning that the subject needs to be well concentrated because the chair movement is very difficult to detect. Introducing a familiarization day before the first testing session (double baseline measurement) might help to minimize the long-term learning effect.

### Effect of 8-week stabilization exercise program on MPT

To the authors’ knowledge, this is the first study to assess the effects of an 8-week stabilization exercise program on MPT. While the patients in the study did show significant improvements in MPT following the exercise program, their improvement was comparable to that of a healthy control group who received no intervention. This suggests that patients’ improvements in MPT may be more attributable to having learned the testing procedure than to the exercise program. Consequently, it is not surprising that we observed no correlation between the change of MPT and the change in the clinical outcomes (NPRS, ODI).

One explanation for the apparent lack of a MPT training effect is that the stabilization program used in this study is not specifically designed to improve lumbar proprioception. These exercises do, however, include some required components (coordination, muscle performance and balance training) to improve proprioception [27]. These results may also be related to the fact that the patients with LBP in this study did not show MPT impairments at the beginning of the program, relative to the control group. It is also possible that different findings would have emerged if we had used a measure of active joint position sense, rather than passive MPT. Lumbar stabilization exercises may provide a better training stimulus for proprioception in situations in which the trunk muscles are involved in maintaining lumbar stability.

### Between-group comparisons on MPT

There is still considerable discrepancy in the literature with regards to impairment of trunk proprioception in patients with LBP [20]. Considering that fundamental differences exist in the different available proprioception measures (MPT, active and passive joint position sense, force sense) [28], we will focus on MPT findings in the transverse plane (lumbar spine rotation) [8, 14, 20].

Previous studies have compared MPT between LBP patients and control subjects, using a device similar to the one used in the present study. Two studies showed significantly higher MPT in patients with LBP [8, 14], while one study showed no difference as in the present study [20]. However, the study conducted by Selfies et al. [20] involved young athletes with history of low back injury at the start of the study, which differ from patients with LBP in the present study. As patients with non-specific LBP are a very heterogeneous group, such conflicting results are not surprising. Unfortunately, the prevalence of patients with a clinically relevant proprioceptive deficit has never been substantiated with a representative sample of patients. Consequently, the number of patients required to study clinically-relevant subgroups of patients, based on their level of proprioceptive deficits, is unknown. Heterogeneity of MPT scores was not higher in the patient group, comparatively to the control group (Fig. 3), as the change of scores over time. Consequently, the presence of clinical subgroups is not obvious within this small sample of patients. The two studies that looked at sex comparisons showed no difference in position sense [8, 20], which concurs with the present findings.

### Limitations

The test-retest reliability results are limited to healthy subjects. Patients with low back pain could have produced different findings. The healthy “control group” used in the present study does not correspond to a conventional control group composed of patients that have not received any treatment. Consequently, although unlikely, it would be possible that such a patient-based control group would have shown no learning of the motion perception measurement, which in turn would have enhanced the likelihood of detecting a GROUP × DAY interaction. As patients with non-specific LBP are a very heterogeneous group, a larger sample of patients is needed to explore possible clinical subgroups based on their level of proprioceptive deficits. This would have been valuable to study relationships with changes in clinical outcomes over treatment. There was no blinding in the assessment to which group the participant was in (LBP patient or control subject), although this type of objective assessment is not believed to be affected by the experimenter. Finally, a single baseline measurement was carried out. Future studies should considered multiple baseline measurements in lumbar MPT in order to wash-out learning effects to better isolate treatment effects.

## Conclusion

Patients with LBP did not show lumbar MPT impairments in the present study, which likely explain why they didn’t improve their proprioception over an 8-week stabilization exercise program, when compared with the changes shown by a healthy control group that not received any treatment. The presence in our data of a long-term learning effect in MPT, with repeated testing, highlights the need to better determine the measurement properties (reliability, validity, responsiveness) of tools used to assess the effects of lumbar stabilization programs.

## Additional file

Additional file 1:
**Description of the 8-week lumbar stabilization exercise program.** (PDF 901 kb)
