# Supplementary material for: The effects of an 8-week stabilization exercise program on lumbar movement sense in patients with low back pain
Source: BMC Musculoskelet Disord. 2016 Jan 14;17:23. doi: 10.1186/s12891-016-0875-4 (PMC4712498; doi:10.1186/s12891-016-0875-4)
Supplement: Additional file 1: — Description of the 8-week lumbar stabilization exercise program. (PDF 901 kb) [file 12891_2016_875_MOESM1_ESM.pdf]

Electronic Supplementary File

Description of the 8-week lumbar stabilization exercise program

From the article entitled: The effects of an 8-week stabilization exercise program on lumbar movement sense in patients with chronic low back pain

Journal name: BMC Musculoskeletal Disorders

Authors: Jean-Alexandre Boucher, D.C., Ph.D.[1,6*], Richard Preuss, P.T., Ph.D.[2,6], Sharon M. Henry, P.T., Ph.D.[3], Jean-Pierre Dumas, M. Sc.[4], Christian Larivière, Ph.D.[5,6]

[1] School of Rehabilitation, Faculty of Medicine, Université de Montréal, Montréal, Québec, Canada

[2] School of Physical & Occupational Therapy, McGill University, Montréal, Québec, Canada

[3] Deparment of Rehabilitation and Movement Science, The University of Vermont, Burlington, Vermont, United States

[4] School of Rehabilitation, Faculty of Medicine, Université de Sherbrooke, Sherbrooke, Québec, Canada

[5] Occupational Health and Safety Research Institute Robert-Sauvé, Montréal, Québec, Canada

[6] Center for Interdisciplinary Research in Rehabilitation of Greater Montreal (CRIR), Montreal Rehabilitation Institute, Montreal, Québec, Canada

* Corresponding author at: (J-A Boucher) Occupational Health and Safety Research Institute Robert-Sauvé, Montréal, Québec, Canada. Tel.: 514-288-1551 #375; Fax: 514-288-6097. E-mail address: charljea@uqtr.ca

This file describes the exercises technical execution and the criteria needed to move from one phase to another. Physiotherapists were involved in the recruitment of patients and supervised the exercise program. They performed their own initial clinical assessment, independently of the clinical and laboratory assessments carried out by the research assistant, to apply their clinical judgment (adjustment of the dosage of the exercise program). In order to standardize the information given to the patients about their condition the pamphlet entitled ‘’Back Book’’ was handed out to the patients as the first visit [1]. The exercise program consisted of the following three phases, with their objectives:

Phase I: Pain management and motor control of deep muscles (2 weeks max)

- More specific patient education was on pain control modalities, anatomy, lumbar stability mechanisms (role of deep trunk muscles). The patient was then required to explain the concepts learned;
- Some stretching exercises (if necessary) and heat/cold application (if necessary);
- Initiation of motor control exercises (see table on next page) in prone position and gradually during overloading exercises (one exercise per category: flexion, extension, oblique). At this stage, emphasis was placed on motor control exercises and exercises prescribed at home were precisely oriented in this direction (daily);
- Feedback during motor control exercises. In clinical practice, the therapist used pressure biofeedback unit to give feedback on the control of lumbar lordosis during abdominal drawing-in maneuver (ADIM). He also proceeded with abdominal palpation to indicate to the patient when superficial abdominal muscles (external oblique and rectus abdominis) were too contracted. At home, we simply advised to reproduce what had been learned and focusing on maintaining a neutral posture of the lumbar spine during the exercises.

Phase II: Beginning impairment and functional level (initiation of exercises with emphasis on quality of movement control)

- Able to complete few repetitions properly (co-contraction and breathing);
- Able to complete repetitions properly (co-contraction and breathing), with a decrease in symptoms during functional activities previously identified as painful with the use of the patient's specific functional scale [2];
- Quality and ease of exercises or movements increase during functional activities.

Phase III: Moderate/Advanced impairment and functional level (endurance development with emphasis on quantity)

- Able to significantly increase the number of repetitions properly (co-contraction and breathing);
- Symptoms’ reproduction during functional activities is minimal (< 2/10 on a pain scale);
- Quality and ease of exercises or good movements during functional activities.

The above-mentioned objectives, for each program phase, had to be successful, at least most of them, so that physiotherapists could make the decision to progress the patient to the next phase.

<u>Lumbar stabilization key concept</u>

For all exercises

1. Adopt a normal breathing (never hold your breath)
2. The part of the exercise involving a movement or support of a position must be performed while exhaling.

**Motor control exercises**	**Key points**	**Dose/Progression**
Pelvic tilt	Flatten lower back to the floor or wall	5-10 sec. hold in neutral 10 reps through pain free range. Progress to pelvic tilt with controlled breathing
Abdominal drawing-in maneuver (ADIM)	Pull the navel in toward the spine and then to the head (‘’J’’ movement)	Able to perform 30 sec. hold on the left and right sides with normal breathing
Multifidus (prone over pillow at lower abdomen)	Swell lower back muscles	10 sec. hold × 10 reps.
**Overload exercises (category)**	**Key principle (illustrations in the following pages)**	
Curl-ups (flexion)	ADIM + Hold shoulder blades off the ground (or mattress)	
Heel Slides (flexion)	ADIM + Swell lower back muscles	
Seated hip flexion (flexion)	ADIM + Maintain lumbar region in neutral position	
Dead Bug (flexion)	ADIM + Hold the position with the arm and the opposite leg just above the horizontal	
Birddog (extension)	ADIM + Elevate the arm and opposite leg while maintaining lumbar region in neutral position	
Side-bridge (oblique)	ADIM + Swell lower back muscles + Elevate hips and maintain the body in straight position	
Bridge (extension)	ADIM + Elevate hips and maintain the body in upright position	
Standing Theraband Exercises (extension)	ADIM + Swell lower back muscles + Maintain lumbar region in neutral position while performing the upper body exercise	

For each overload exercise, three or four levels of difficulty allowed a progression, as illustrated in the following pages. A 2-page document including these illustrations allowed the physiotherapist to identify (checkboxes), each week, the exercises to perform at home. It was also possible to clearly identify the dosage [frequency (per week), number of repetitions]. At home or at the clinic, the therapist and the patient chose the equipment to create an unstable surface. The patient was asked to get this equipment at home, as usual when following an exercise program in physiotherapy clinics.

Guidelines for the post-treatment home exercise program (after 8 weeks of physiotherapy)

During the last appointment, the physiotherapist had to review the home exercise program with the patient to ensure that he knew and understood the expected exercise and frequency, so that he could continue the exercises once the treatment ended at 8 weeks.

Instruction for the functional activities	• The patient should continue during the day with changes in daily activities taught by the physiotherapist.
Specific exercises	• Deep muscle co-contraction, if necessary, during daily activities and/or in separate exercises<br>• Overload exercises (at least one of flexion, extension and oblique) for a total of 20-30 minutes at a rate of 3 times a week.
General advices	• Encourage the patient to (1) continue to be active, (2) respect throughout the day changes in daily activities and (3) do specific overload exercises.

I. Flexion category

A. Curl-up

Phase	Image	Patient Instructions: Key Points	Dosage
I	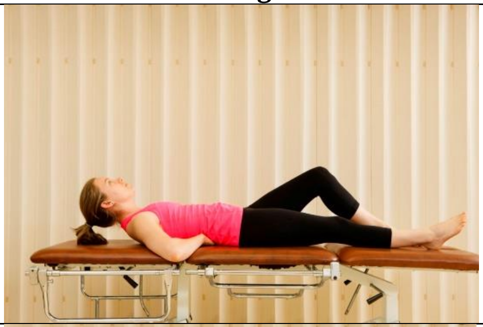	Patient lies supine with both hands positioned under the lumbar spine supporting a neutral curve, both elbows on the mat, hip and knee flexed on one side and extended on the opposite, performs ADIM – patient instructed to lift the shoulder blades off the mat while maintaining a neutral neck position.	Hold 5 seconds × 10 reps.
IIa	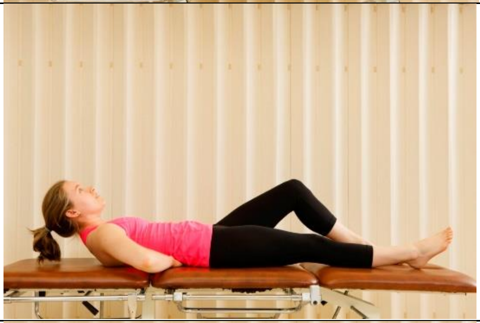	Instruct patient to lift both elbows off of the mat while performing previously described curl-up.	Hold 5 seconds × 10 reps.
IIb	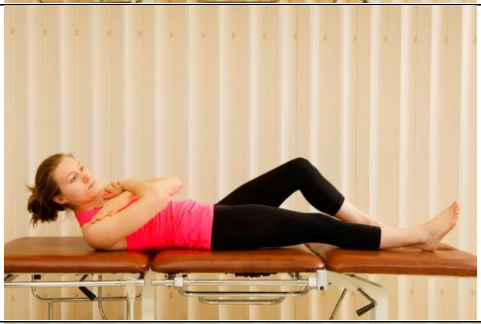	Patient lies supine with hip and knee flexed on one side and extended on the opposite side, hands across chest. Patient instructed to perform ADIM, curl-up lifting head and shoulders off mat with rotation to the right, return and rotate to the left.	Hold 5 seconds × 10 reps. on each side
III	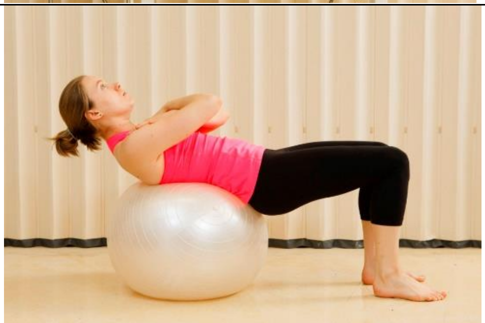	Patient performs phase I or phase II curl-up on unstable surface.	Hold 5 seconds × 20 reps.

B. Dead-Bug

Phase	Image	Patient Instructions: Key Points	Dosage
I	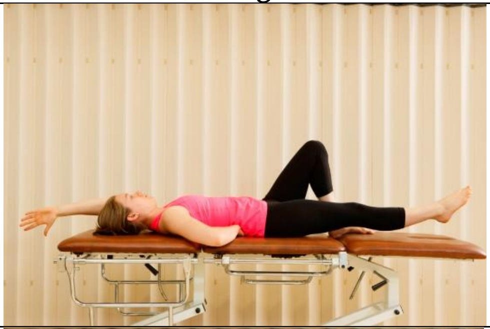	Patient lies supine with right hand positioned under the lumbar spine – hips, knees flexed to 90°, and left arm at side. Patient instructed to perform ADIM and slowly extend right lower extremity and lift left arm overhead until both arm and leg are just above horizontal – slightly elevate off the mat.	Hold 5 seconds and returns slowly to start position × 10 reps. Repeat on opposite side.
IIa	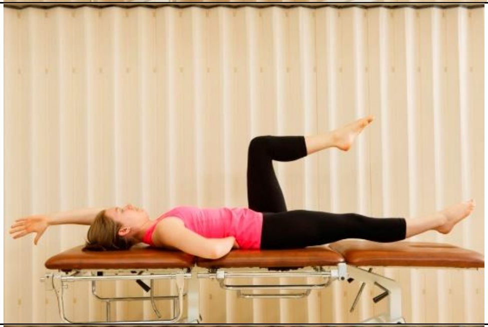	Repeat phase I exercise with large amplitude/slow motion at hip and shoulder, continue on same side until patient fatigues/is unable to properly perform exercise – return to start position and repeat on opposite sides. Motion should be at hip and shoulder; not torso.	× 10 reps. on each side
IIb	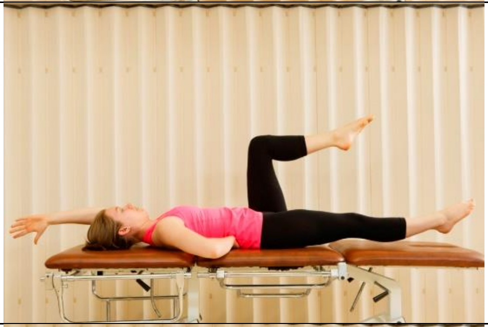	Repeat phase IIa exercise with small amplitude/fast motion	× 10 reps. on each side
III	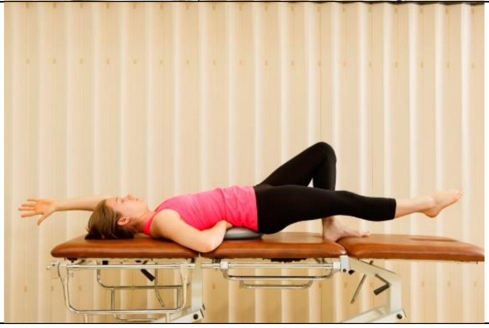	Patient performs phase I or phase II dead-bug on unstable surface.	× 20 reps. on each side

C. Heel Slide

Phase	Image	Patient Instructions: Key Points	Dosage
I	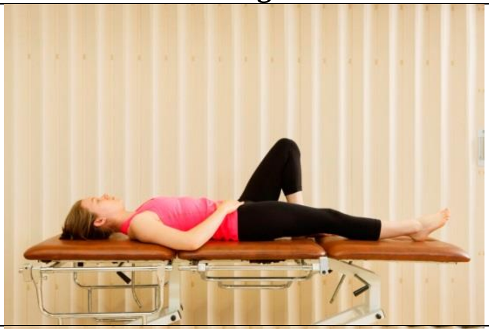	Patient lies supine in hooklying position. Performs co-contractions, slides one heel out until knee is straight and then returns. Repeats on opposite side. Palpate medial to ASIS to verify transverse abdominis contraction.	× 30 reps total maintaining co-contraction
I	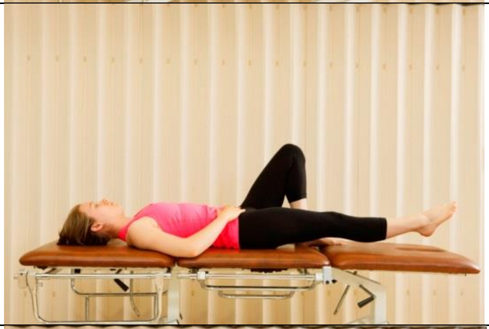	Patient lies supine in hooklying position. Performs co-contractions, extends one knee so heel of foot is 3-4 inches above table surface. Hold for 2 seconds. Return to start position and repeat on opposite side. Palpate medial to ASIS to verify transverse abdominis contraction.	× 30 reps total maintaining co-contraction
IIa	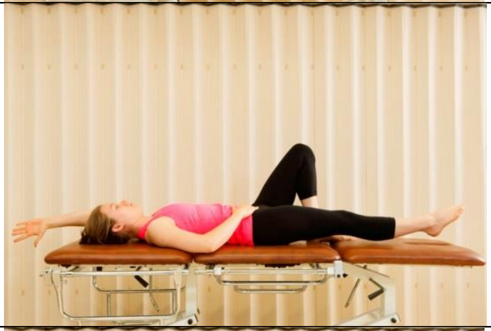	Patient lies supine in hooklying position. Performs co-contractions, extends one knee so heel of foot is 3-4 inches above table surface and simultaneously flexes opposite shoulder so hand is 3-4 inches above table surface. Hold for 2 seconds. Return to start position and repeat on opposite side. Palpate medial to ASIS to verify transverse abdominis contraction.	× 30 reps total maintaining co-contraction
IIb	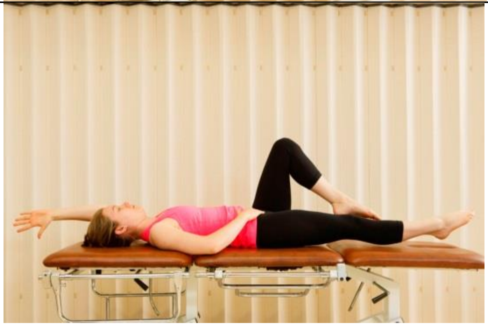	Patient lies supine in hooklying position. Performs co-contractions and lift both 3 feet inches off the table surface. Extends one knee so heel of foot is 3-4 inches above table surface and simultaneously flexes opposite shoulder so hand is 3-4 inches above table surface. Hold for 2 seconds. Return to start position and repeat on opposite side. Palpate medial to ASIS to verify transverse abdominis contraction.	× 30 reps total maintaining co-contraction
III	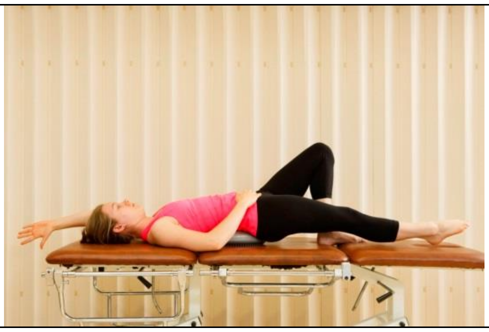	Patient perform phase I or phase II heel slide on unstable surface.	30 reps total

D. Seated Hip Flexion

Phase	Image	Patient Instructions: Key Points	Dosage
I	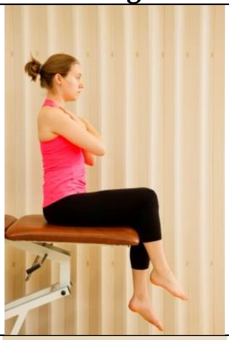	Patient sits on stable surface with spine in neutral position. Patient instructed to perform ADIM while slowly flexing one hip raising flexed knee > 4 inches off table surface. Repeat on opposite side. Patient must maintain neutral spine and transversus abdominis contraction.	× 30 reps on each side
II	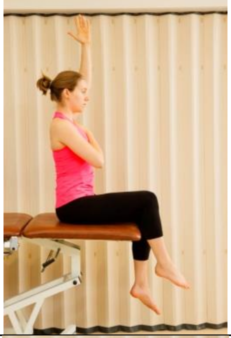	Patient sits on stable surface with spine in neutral position. Patient instructed to perform ADIM while slowly flexing one hip raising flexed knee > 4 inches off table surface and simultaneously flexing opposite shoulder. Repeat on opposite side. Patient must maintain neutral spine and transversus abdominis contraction.	× 30 reps on each side
IIIa	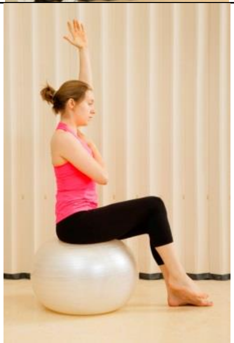	Patient sits on unstable surface such as physioball or dynadisc with spine in neutral position. Patient instructed to perform ADIM while slowly flexing one hip raising flexed knee > 4 inches off table surface and simultaneously flexing opposite shoulder. Repeat on opposite side. Patient must maintain neutral spine and transverse abdominis contraction.	× 30 reps on each side
IIIb	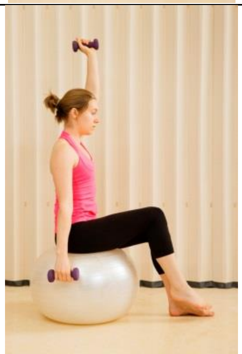	Patient sits on unstable surface such as physioball or dynadisc with spine in neutral position and holds a small weight in each hand. Patient instructed to perform ADIM while slowly flexing one hip raising flexed knee > 4 inches off table surface and simultaneously flexing opposite shoulder. Repeat on opposite side. Patient must maintain neutral spine and transverse abdominis contraction.	× 30 reps on each side
IIIc		Patient sits on unstable surface such as physioball or dynadisc with spine in neutral position. Patient instructed to perform ADIM and then performs resisted exercises using tubing. Patient must maintain neutral spine and transverse abdominis contraction.	× 30 reps on each side

II. Extension category

A. Birddog

Phase	Image	Patient Instructions: Key Points	Dosage
I	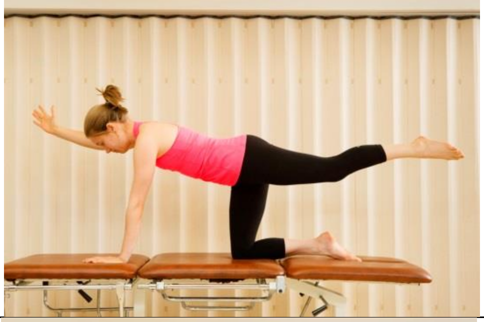	Patient positioned in quadruped and instructed to perform ADIM and elevate one arm progress to one leg - parallel to mat. Patient’s spine must remain neutral without rotation, shift, or sidebend. Hold 5 seconds and slowly return to start position and repeat on opposite side.	× 30 reps. on each side
IIa	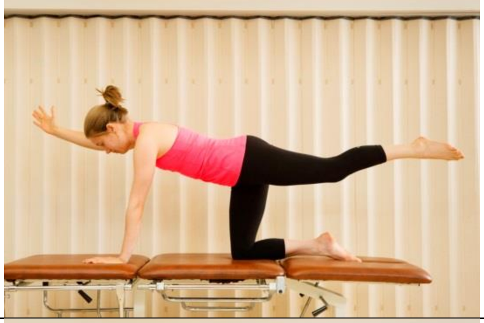	Patient in quadruped position and instructed to perform ADIM while simultaneously elevating contralateral upper extremity / lower extremity. Patient’s spine must remain neutral without rotation, shift, or sidebend. Hold 5 seconds and slowly return to start position. Use palpation to verify co-contraction.	× 30 reps. on each side
IIb	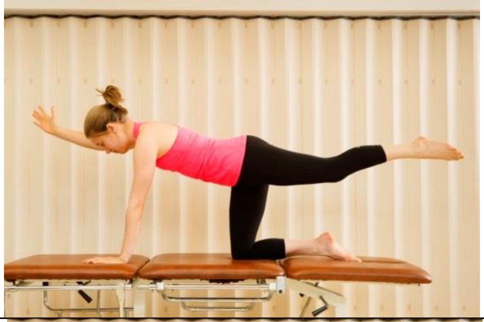	Patient to assume elevated birddog position and perform ADIM. Patient instructed to draw squares with hand and foot while limiting motion to shoulder and hip. Patient’s spine must remain neutral without rotation, shift, or sidebend.	× 30 reps. on each side
III	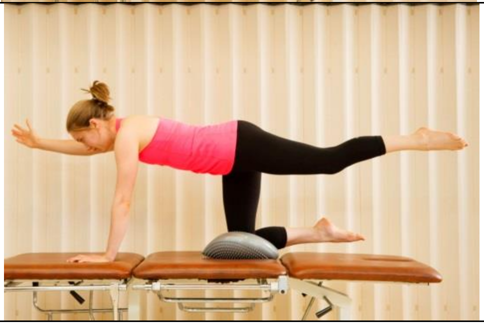	Patient performs either phase II birddog with unstable surface under knees.	× 30 reps. on each side

B. Bridge

Phase	Image	Patient Instructions: Key Points	Dosage
I	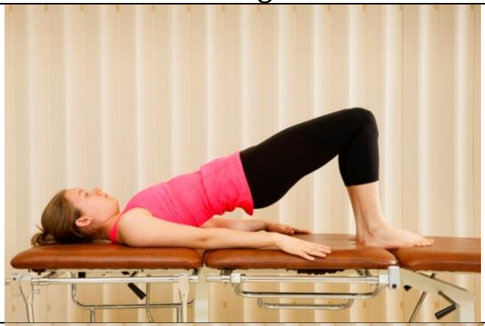	Patient lies in supine hooklying position. Patient instructed to perform ADIM and then bridge and hold for 5 seconds. Palpate to verify transversus abdominis contraction.	× 30 reps.
IIa	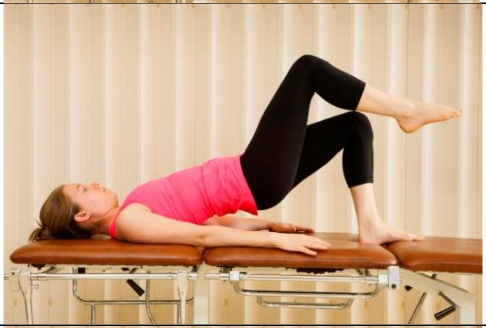	Patient lies in supine hooklying position. Patient instructed to perform ADIM and bridge. Patient instructed to march in place alternately lifting right and left foot. A dowel may be placed across ASISs to monitor pelvic movement.	× 30 reps. on each side
IIb	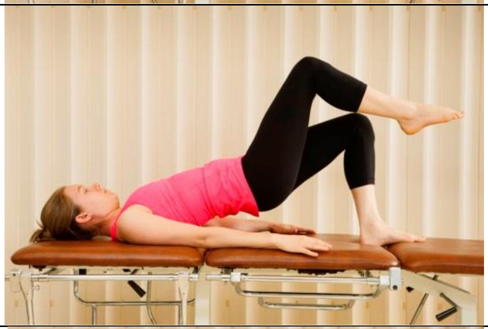	Patient lies in supine hooklying position, lift one foot off table surface. Patient instructed to perform ADIM and then bridge with one leg. Hold for 2 seconds. Return to hooklying and repeat on opposite side.	× 30 reps. on each side
III	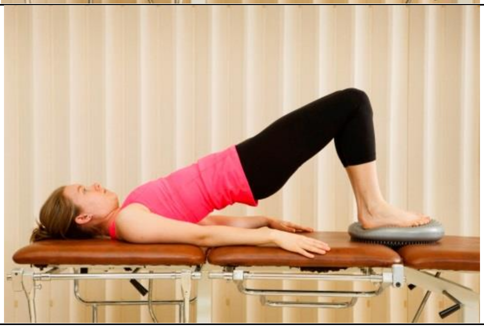	Patient instructed to perform phase I or phase II bridge with unstable surface under feet.	× 30 reps.

III. Oblique category

A. Side-Bridge

Phase	Image	Patient Instructions: Key Points	Dosage
I	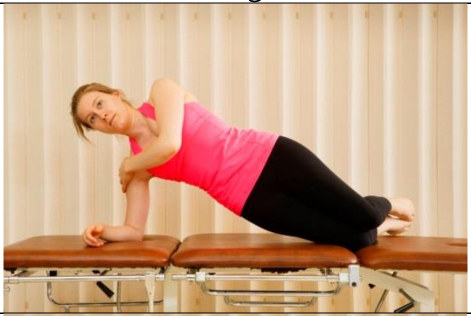	Patient sidelying supported by elbow and hip – elbow at 90°, knees flexed and hips in neutral position. Patient instructed to perform ADIM, elevate hips off the mat – transferring weight from hips to knees. Place hand over the contralateral deltoid. Phase I progression – perform this exercise with free hand on ipsilateral hip.	Hold for 5 sec × 30 reps. Repeat on opposite side.
IIa	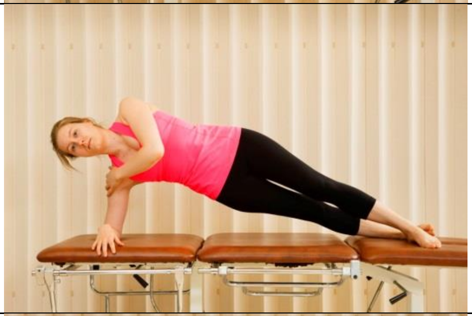	Patient instructed to place top foot in front of the lower foot, hips in neutral position and knees extended – patient instructed to perform ADIM, transferring weight to their feet and elbow while elevating hips. Place hand over contralateral deltoid and progressing to ipsilateral hip.	Hold for 3 seconds × 30 reps. Repeat on opposite side.
IIb	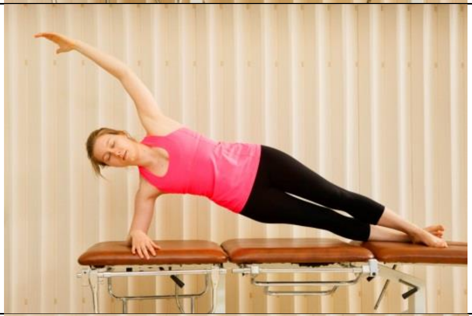	Patient instructed to place top foot in front of the lower foot, hips in neutral position and knees extended – patient instructed to perform ADIM, transferring weight to their feet and elbow while elevating hips. Patient instructed to hold position while abducting shoulder and raising arm overhead.	Hold for 3 seconds × 30 reps. Repeat on opposite side.
IIc	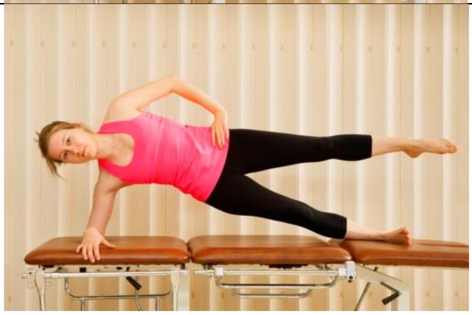	Patient instructed to place top foot in front of the lower foot, hips in neutral position and knees flexed – patient instructed to place free hand on hip, perform ADIM, transferring weight to their knees and elbow while elevating hips. Patient instructed to hold position while abducting free hip raising leg 18’’ above opposite leg. Progress this exercise with hips and knees extended.	Hold for 3 seconds × 30 reps. Repeat on opposite side.
III	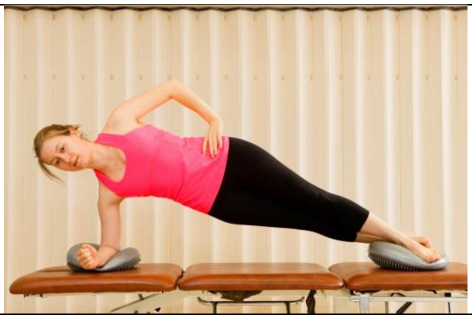	Patient performs phase I or phase II side-bridge with unstable surface elbow, feet or both.	× 30 reps.

IV. Standing Options

A. Standing Theraband Exercises

Phase	Image	Patient Instructions: Key Points	Dosage
I	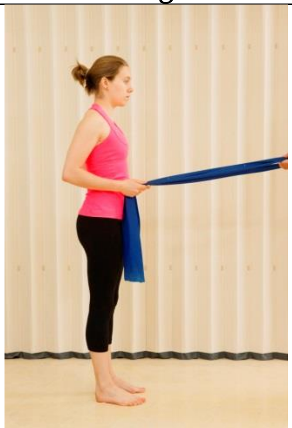	Patient stands with spine in neutral position. Patient instructed to perform ADIM while holding theraband and performing rowing or scapular retraction exercise.	× 30 reps with neutral spine and transversus abdominis contraction
II	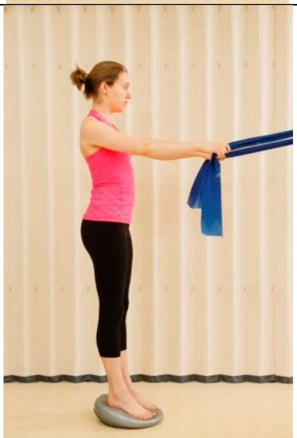	Patient stands with spine in neutral position while standing on an unstable surface such as a dynadisc or wobble board. Patient instructed to perform ADIM while holding theraband and performing rowing or scapular retraction exercise.	× 30 reps with neutral spine and transversus abdominis contraction
III	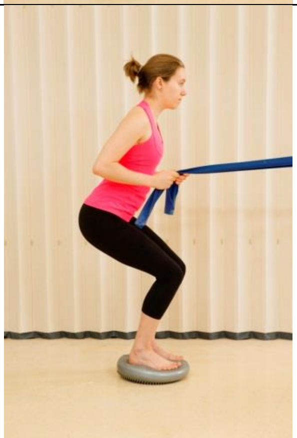	Patient instructed to perform phase II standing exercise with hips and knees bent – patient instructed to squat keeping the knees behind the toes.	× 30 reps with neutral spine and transversus abdominis contraction

References

1. Burton AK, Waddell G, Tillotson KM, Summerton N. Information and advice to patients with back pain can have a positive effect. A randomized controlled trial of a novel educational booklet in primary care. Spine. 1999; 24:2484-2491.

2. Stratford P, Gill C, Westaway M, Binkley J. Assessing disability and change on individual patients: A report of a patient specific measure. Physiotherapy Canada. 1995;47:258-263.
